# 1-Chloro-1-[(4-methyl­phen­yl)hydrazinyl­idene]propan-2-one

**DOI:** 10.1107/S1600536811026419

**Published:** 2011-07-09

**Authors:** Abdullah M. Asiri, Abdulrahman O. Al-Youbi, Mohie E. M. Zayed, Seik Weng Ng

**Affiliations:** a Chemistry Department, Faculty of Science, King Abdulaziz University, PO Box 80203 Jeddah, Saudi Arabia; b The Center of Excellence for Advanced Materials Research, King Abdul Aziz University, PO Box 8020 Jeddah, Saudi Arabia; c Department of Chemistry, University of Malaya, 50603 Kuala Lumpur, Malaysia

## Abstract

The asymmetric unit of the title compound, C_10_H_11_ClN_2_O, contains two mol­ecules. The non-H atoms of each mol­ecule lie approximately on a plane (r.m.s. deviations = 0.062 and 0.110 Å), and the C=N double bond has a *Z*-configuration in both independent mol­ecules. In the crystal, adjacent mol­ecules are linked by N—H⋯O_carbon­yl_ hydrogen bonds, forming chains running along [100].

## Related literature

For the synthesis, see: Benincori *et al.* (1990 ▶); Sayed *et al.* (2002 ▶). For background to the title compound, see: Asiri *et al.* (2010 ▶).
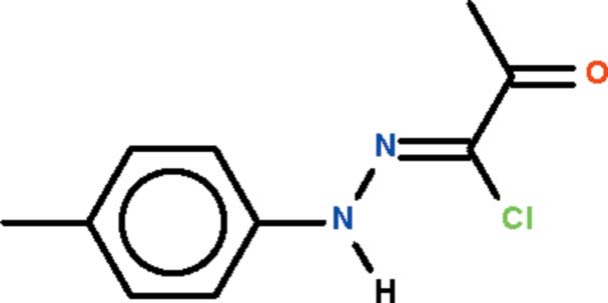


## Experimental

### 

#### Crystal data

C_10_H_11_ClN_2_O
*M* *_r_* = 210.66
Monoclinic, 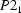

*a* = 11.0572 (3) Å
*b* = 7.6570 (2) Å
*c* = 12.4613 (3) Å
β = 105.063 (3)°
*V* = 1018.79 (5) Å^3^
*Z* = 4
Cu *K*α radiation
μ = 3.06 mm^−1^
*T* = 100 K
0.20 × 0.02 × 0.02 mm

#### Data collection

Agilent SuperNova Dual diffractometer with an Atlas detector
Absorption correction: multi-scan (*CrysAlis PRO*; Agilent, 2010 ▶) *T*_min_ = 0.580, *T*_max_ = 0.941
3467 measured reflections
2667 independent reflections
2516 reflections with *I* > 2σ(*I*)
*R*_int_ = 0.021

#### Refinement

*R*[*F* ^2^ > 2σ(*F* ^2^)] = 0.045
*wR*(*F* ^2^) = 0.125
*S* = 1.06
2667 reflections
266 parameters
1 restraint
H atoms treated by a mixture of independent and constrained refinement
Δρ_max_ = 0.31 e Å^−3^
Δρ_min_ = −0.42 e Å^−3^
Absolute structure: Flack (1983 ▶), 533 Friedel pairs
Flack parameter: 0.17 (2)

### 

Data collection: *CrysAlis PRO* (Agilent, 2010 ▶); cell refinement: *CrysAlis PRO*; data reduction: *CrysAlis PRO*; program(s) used to solve structure: *SHELXS97* (Sheldrick, 2008 ▶); program(s) used to refine structure: *SHELXL97* (Sheldrick, 2008 ▶); molecular graphics: *X-SEED* (Barbour, 2001 ▶); software used to prepare material for publication: *publCIF* (Westrip, 2010 ▶).

## Supplementary Material

Crystal structure: contains datablock(s) global, I. DOI: 10.1107/S1600536811026419/xu5262sup1.cif

Structure factors: contains datablock(s) I. DOI: 10.1107/S1600536811026419/xu5262Isup2.hkl

Supplementary material file. DOI: 10.1107/S1600536811026419/xu5262Isup3.cml

Additional supplementary materials:  crystallographic information; 3D view; checkCIF report

## Figures and Tables

**Table 1:** Hydrogen-bond geometry (Å, °)

*D*—H⋯*A*	*D*—H	H⋯*A*	*D*⋯*A*	*D*—H⋯*A*
N2—H2⋯O2	0.88 (5)	2.12 (5)	2.975 (4)	162 (4)
N4—H4⋯O1^i^	0.83 (5)	2.12 (5)	2.909 (4)	159 (4)

Symmetry code: (i) 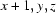.

**Fig. 1.**
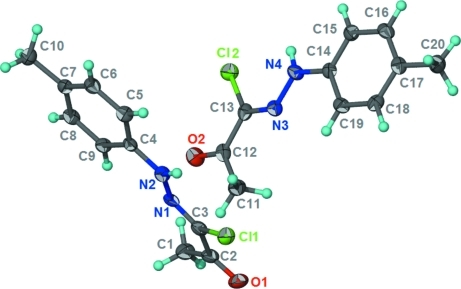
Thermal ellipsoid plot (Barbour, 2001) of the two independent molecules of C10H11ClN2O at the 70% probability level; hydrogen atoms are drawn as spheres of arbitrary radius.

## supplementary crystallographic information

### Comment

We have previously reported the synthesis of ethyl (*Z*)-2-chloro-2-(2-phenylhydrazin-1-ylidene) acetate by the reaction of benzenediazonium chloride with ethyl 2-chloro-3-oxobutanoate (Asiri *et al.*, 2010). The compound is an ester. In the present study, the use of a substituted benzenediazonium chloride and the methyl ester (instead of the ethyl ester) afforded a 1-chloro-1-(arylhydrazono)-2-propanone. Such ketones are intermediates in the synthesis of pyrazoles (Sayed *et al.*, 2002) and other heterocycles (Benincori *et al.*, 1990). In the 4-methyl substituted compound (Scheme I, Fig. 1), the non-hydrogen atoms lie on a plane [r.m.s. deviation 0.062 and 0.110 Å in the two independent molecules]. (Scheme I, Fig. 1). The C_aryl_–N(H)–N═ C(S)═O portion adopts an extended zigzag conformation. Adjacent molecules are linked by an *N*–*H*···O_carbonyl_ hydrogen bond to form a chain running [1 0 0].

### Experimental

To a stirred solution of methyl 2-chloro-3-oxobutanoate (1.64 g, 10 mmol) in ethanol (100 ml) was added sodium acetate trihydrate (1.30 g, 10 mmol). The mixture was chilled to 273 K and then treated with a cold solution of *p*-nitrobenzenediazonium chloride, prepared by diazotizing *p*-methylaniline (1.07 g, 10 mmol) dissolved in 6*M* hydrochloric acid (6 ml) with a solution of sodium nitrite (0.70 g, 10 mmol) in water (10 ml). The addition of the diazonium salt solution was carried out with rapid stirring over a period of 20 min. The reaction mixture was stirred for further 15 min. and left for 3 h in refrigerator. The resulting solid was collected by filtration and washed thoroughly with water. The crude product was crystallized from ethanol to give the corresponding hydrazonoyl chloride.

### Refinement

Carbon-bound H-atoms were placed in calculated positions [C—H 0.95 to 0.98 Å, *U*_iso_(H) 1.2 to 1.5*U*_eq_(C)] and were included in the refinement in the riding model approximation.

The amino H-atom was located in a difference Fourier map, and was freely refined.

The Flack parameter was refined from 533 Friedel pairs.

Omitted from the refinement was the (2 - 2 2) reflection.

### Crystal data

**Table:**

C_10_H_11_ClN_2_O	*F*(000) = 440
*M**_r_* = 210.66	*D*_x_ = 1.373 Mg m^−^^3^
Monoclinic, *P*2_1_	Cu *K*α radiation, λ = 1.54184 Å
Hall symbol: P 2yb	Cell parameters from 1695 reflections
*a* = 11.0572 (3) Å	θ = 3.7–74.0°
*b* = 7.6570 (2) Å	µ = 3.06 mm^−^^1^
*c* = 12.4613 (3) Å	*T* = 100 K
β = 105.063 (3)°	Prism, yellow
*V* = 1018.79 (5) Å^3^	0.20 × 0.02 × 0.02 mm
*Z* = 4	

### Data collection

**Table:**

Agilent SuperNova Dual diffractometer with an Atlas detector	2667 independent reflections
Radiation source: SuperNova (Cu) X-ray Source	2516 reflections with *I* > 2σ(*I*)
Mirror	*R*_int_ = 0.021
Detector resolution: 10.4041 pixels mm^-1^	θ_max_ = 74.2°, θ_min_ = 3.7°
ω scan	*h* = −13→7
Absorption correction: multi-scan (*CrysAlis PRO*; Agilent, 2010)	*k* = −9→9
*T*_min_ = 0.580, *T*_max_ = 0.941	*l* = −13→15
3467 measured reflections	

### Refinement

**Table:**

Refinement on *F*^2^	Secondary atom site location: difference Fourier map
Least-squares matrix: full	Hydrogen site location: inferred from neighbouring sites
*R*[*F*^2^ > 2σ(*F*^2^)] = 0.045	H atoms treated by a mixture of independent and constrained refinement
*wR*(*F*^2^) = 0.125	*w* = 1/[σ^2^(*F*_o_^2^) + (0.0867*P*)^2^ + 0.3419*P*] where *P* = (*F*_o_^2^ + 2*F*_c_^2^)/3
*S* = 1.06	(Δ/σ)_max_ = 0.001
2667 reflections	Δρ_max_ = 0.31 e Å^−^^3^
266 parameters	Δρ_min_ = −0.42 e Å^−^^3^
1 restraint	Absolute structure: Flack (1983), 533 Friedel pairs
Primary atom site location: structure-invariant direct methods	Flack parameter: 0.17 (2)

### Fractional atomic coordinates and isotropic or equivalent isotropic displacement parameters (Å^2^)

**Table:**

	*x*	*y*	*z*	*U*_iso_*/*U*_eq_	
Cl1	0.22799 (7)	0.50001 (13)	0.90675 (6)	0.0252 (2)	
Cl2	0.75288 (7)	0.52179 (14)	1.00248 (6)	0.0252 (2)	
N1	0.2864 (2)	0.4827 (4)	1.1276 (2)	0.0187 (6)	
N2	0.3920 (3)	0.4043 (4)	1.1235 (2)	0.0195 (6)	
H2	0.416 (4)	0.391 (7)	1.062 (4)	0.035 (13)*	
N3	0.7037 (2)	0.4842 (4)	0.7819 (2)	0.0193 (6)	
N4	0.8068 (3)	0.5762 (4)	0.7850 (2)	0.0201 (6)	
H4	0.853 (5)	0.615 (7)	0.844 (4)	0.035 (13)*	
O1	0.0213 (2)	0.6872 (4)	0.9612 (2)	0.0297 (7)	
O2	0.5199 (3)	0.3196 (4)	0.9474 (2)	0.0281 (6)	
C1	0.0693 (3)	0.6493 (6)	1.1582 (3)	0.0253 (8)	
H1A	0.0074	0.7420	1.1556	0.038*	
H1B	0.1477	0.6800	1.2125	0.038*	
H1C	0.0373	0.5391	1.1800	0.038*	
C2	0.0928 (3)	0.6290 (6)	1.0454 (3)	0.0225 (8)	
C3	0.2071 (3)	0.5347 (5)	1.0390 (3)	0.0207 (7)	
C4	0.4745 (3)	0.3415 (5)	1.2216 (3)	0.0177 (7)	
C5	0.5915 (3)	0.2783 (5)	1.2169 (3)	0.0207 (7)	
H5	0.6156	0.2824	1.1491	0.025*	
C6	0.6723 (3)	0.2097 (5)	1.3117 (3)	0.0212 (7)	
H6	0.7515	0.1664	1.3077	0.025*	
C7	0.6406 (3)	0.2026 (5)	1.4124 (3)	0.0200 (7)	
C8	0.5243 (3)	0.2683 (5)	1.4160 (3)	0.0205 (8)	
H8	0.5011	0.2654	1.4842	0.025*	
C9	0.4416 (3)	0.3377 (5)	1.3228 (3)	0.0190 (7)	
H9	0.3630	0.3825	1.3274	0.023*	
C10	0.7273 (3)	0.1199 (6)	1.5139 (3)	0.0255 (8)	
H10A	0.7083	0.1659	1.5810	0.038*	
H10B	0.8143	0.1473	1.5154	0.038*	
H10C	0.7155	−0.0070	1.5108	0.038*	
C11	0.4814 (4)	0.2920 (5)	0.7503 (3)	0.0240 (8)	
H11A	0.4059	0.2297	0.7562	0.036*	
H11B	0.5335	0.2139	0.7190	0.036*	
H11C	0.4573	0.3938	0.7019	0.036*	
C12	0.5538 (3)	0.3510 (5)	0.8636 (3)	0.0203 (8)	
C13	0.6703 (3)	0.4509 (5)	0.8711 (3)	0.0194 (7)	
C14	0.8352 (3)	0.6173 (5)	0.6841 (3)	0.0176 (7)	
C15	0.9434 (3)	0.7145 (5)	0.6877 (3)	0.0206 (7)	
H15	0.9979	0.7478	0.7569	0.025*	
C16	0.9699 (3)	0.7618 (5)	0.5883 (3)	0.0220 (8)	
H16	1.0423	0.8302	0.5907	0.026*	
C17	0.8940 (3)	0.7123 (5)	0.4861 (3)	0.0204 (7)	
C18	0.7876 (3)	0.6112 (5)	0.4846 (3)	0.0209 (7)	
H18	0.7349	0.5739	0.4154	0.025*	
C19	0.7581 (3)	0.5651 (5)	0.5822 (3)	0.0195 (7)	
H19	0.6853	0.4977	0.5796	0.023*	
C20	0.9210 (4)	0.7668 (6)	0.3778 (3)	0.0263 (8)	
H20A	0.8425	0.7988	0.3241	0.040*	
H20B	0.9602	0.6695	0.3484	0.040*	
H20C	0.9778	0.8673	0.3910	0.040*	

### Atomic displacement parameters (Å^2^)

**Table:**

	*U*^11^	*U*^22^	*U*^33^	*U*^12^	*U*^13^	*U*^23^
Cl1	0.0246 (4)	0.0350 (5)	0.0168 (4)	−0.0019 (4)	0.0070 (3)	0.0016 (4)
Cl2	0.0246 (4)	0.0320 (5)	0.0193 (4)	−0.0022 (4)	0.0065 (3)	−0.0005 (4)
N1	0.0137 (12)	0.0207 (17)	0.0231 (13)	−0.0041 (12)	0.0074 (11)	−0.0008 (13)
N2	0.0191 (14)	0.0255 (17)	0.0158 (13)	−0.0014 (13)	0.0082 (12)	0.0000 (12)
N3	0.0170 (13)	0.0199 (17)	0.0230 (13)	0.0019 (12)	0.0089 (11)	0.0016 (13)
N4	0.0201 (14)	0.0242 (16)	0.0176 (14)	−0.0045 (12)	0.0079 (12)	−0.0019 (13)
O1	0.0162 (12)	0.046 (2)	0.0253 (14)	−0.0022 (12)	0.0019 (11)	0.0054 (13)
O2	0.0251 (13)	0.0375 (17)	0.0263 (14)	−0.0004 (12)	0.0146 (11)	0.0041 (12)
C1	0.0190 (16)	0.034 (2)	0.0246 (18)	0.0011 (16)	0.0089 (15)	0.0000 (17)
C2	0.0163 (15)	0.030 (2)	0.0212 (17)	−0.0053 (16)	0.0053 (14)	−0.0001 (16)
C3	0.0189 (15)	0.025 (2)	0.0194 (15)	−0.0068 (16)	0.0079 (13)	−0.0007 (15)
C4	0.0165 (16)	0.0152 (17)	0.0218 (17)	−0.0030 (14)	0.0057 (14)	−0.0003 (14)
C5	0.0194 (17)	0.0233 (19)	0.0217 (17)	0.0007 (15)	0.0096 (14)	−0.0010 (15)
C6	0.0189 (16)	0.0219 (17)	0.0245 (18)	−0.0011 (15)	0.0088 (14)	−0.0003 (15)
C7	0.0195 (16)	0.0182 (17)	0.0206 (17)	−0.0037 (14)	0.0024 (14)	0.0001 (14)
C8	0.0214 (17)	0.0245 (19)	0.0181 (16)	−0.0041 (15)	0.0099 (14)	−0.0036 (15)
C9	0.0172 (16)	0.0243 (18)	0.0172 (16)	−0.0021 (14)	0.0072 (14)	−0.0012 (14)
C10	0.0229 (17)	0.027 (2)	0.0252 (18)	0.0014 (16)	0.0043 (15)	0.0060 (16)
C11	0.0217 (18)	0.028 (2)	0.0217 (18)	−0.0005 (16)	0.0042 (15)	−0.0003 (16)
C12	0.0199 (17)	0.0193 (18)	0.0255 (18)	0.0056 (15)	0.0129 (15)	0.0032 (15)
C13	0.0219 (17)	0.0207 (18)	0.0167 (15)	0.0034 (14)	0.0068 (14)	0.0020 (13)
C14	0.0174 (15)	0.0181 (18)	0.0185 (16)	0.0023 (14)	0.0067 (13)	0.0013 (14)
C15	0.0196 (16)	0.0199 (17)	0.0223 (17)	0.0014 (14)	0.0057 (14)	0.0016 (15)
C16	0.0149 (15)	0.0212 (19)	0.032 (2)	0.0006 (15)	0.0104 (15)	0.0019 (16)
C17	0.0218 (17)	0.0204 (17)	0.0220 (17)	0.0023 (15)	0.0108 (14)	0.0014 (15)
C18	0.0198 (16)	0.0244 (19)	0.0186 (16)	0.0049 (15)	0.0050 (14)	0.0002 (14)
C19	0.0171 (15)	0.0189 (18)	0.0245 (16)	0.0000 (13)	0.0087 (14)	−0.0015 (14)
C20	0.0234 (18)	0.035 (2)	0.0237 (18)	0.0013 (17)	0.0109 (15)	0.0076 (17)

### Geometric parameters (Å, °)

**Table:**

Cl1—C3	1.743 (3)	C8—C9	1.384 (5)
Cl2—C13	1.742 (4)	C8—H8	0.9500
N1—C3	1.281 (4)	C9—H9	0.9500
N1—N2	1.326 (4)	C10—H10A	0.9800
N2—C4	1.407 (5)	C10—H10B	0.9800
N2—H2	0.88 (5)	C10—H10C	0.9800
N3—C13	1.283 (4)	C11—C12	1.500 (5)
N3—N4	1.332 (4)	C11—H11A	0.9800
N4—C14	1.409 (4)	C11—H11B	0.9800
N4—H4	0.83 (5)	C11—H11C	0.9800
O1—C2	1.221 (5)	C12—C13	1.480 (5)
O2—C12	1.222 (4)	C14—C19	1.391 (5)
C1—C2	1.502 (5)	C14—C15	1.399 (5)
C1—H1A	0.9800	C15—C16	1.393 (5)
C1—H1B	0.9800	C15—H15	0.9500
C1—H1C	0.9800	C16—C17	1.383 (5)
C2—C3	1.477 (5)	C16—H16	0.9500
C4—C5	1.397 (5)	C17—C18	1.404 (5)
C4—C9	1.399 (5)	C17—C20	1.515 (5)
C5—C6	1.386 (5)	C18—C19	1.385 (5)
C5—H5	0.9500	C18—H18	0.9500
C6—C7	1.389 (5)	C19—H19	0.9500
C6—H6	0.9500	C20—H20A	0.9800
C7—C8	1.391 (5)	C20—H20B	0.9800
C7—C10	1.513 (5)	C20—H20C	0.9800
			
C3—N1—N2	121.3 (3)	H10A—C10—H10B	109.5
N1—N2—C4	120.0 (3)	C7—C10—H10C	109.5
N1—N2—H2	123 (3)	H10A—C10—H10C	109.5
C4—N2—H2	117 (3)	H10B—C10—H10C	109.5
C13—N3—N4	121.1 (3)	C12—C11—H11A	109.5
N3—N4—C14	118.7 (3)	C12—C11—H11B	109.5
N3—N4—H4	123 (3)	H11A—C11—H11B	109.5
C14—N4—H4	118 (3)	C12—C11—H11C	109.5
C2—C1—H1A	109.5	H11A—C11—H11C	109.5
C2—C1—H1B	109.5	H11B—C11—H11C	109.5
H1A—C1—H1B	109.5	O2—C12—C13	120.2 (3)
C2—C1—H1C	109.5	O2—C12—C11	122.5 (3)
H1A—C1—H1C	109.5	C13—C12—C11	117.3 (3)
H1B—C1—H1C	109.5	N3—C13—C12	119.3 (3)
O1—C2—C3	120.3 (3)	N3—C13—Cl2	123.6 (3)
O1—C2—C1	122.3 (3)	C12—C13—Cl2	117.1 (3)
C3—C2—C1	117.4 (3)	C19—C14—C15	119.9 (3)
N1—C3—C2	120.6 (3)	C19—C14—N4	121.6 (3)
N1—C3—Cl1	122.6 (3)	C15—C14—N4	118.5 (3)
C2—C3—Cl1	116.8 (3)	C16—C15—C14	119.0 (3)
C5—C4—C9	119.4 (3)	C16—C15—H15	120.5
C5—C4—N2	118.7 (3)	C14—C15—H15	120.5
C9—C4—N2	121.9 (3)	C17—C16—C15	122.0 (3)
C6—C5—C4	119.6 (3)	C17—C16—H16	119.0
C6—C5—H5	120.2	C15—C16—H16	119.0
C4—C5—H5	120.2	C16—C17—C18	118.0 (3)
C5—C6—C7	121.8 (3)	C16—C17—C20	122.1 (3)
C5—C6—H6	119.1	C18—C17—C20	119.9 (3)
C7—C6—H6	119.1	C19—C18—C17	121.1 (3)
C6—C7—C8	117.8 (3)	C19—C18—H18	119.5
C6—C7—C10	121.1 (3)	C17—C18—H18	119.5
C8—C7—C10	121.0 (3)	C18—C19—C14	120.0 (3)
C9—C8—C7	121.8 (3)	C18—C19—H19	120.0
C9—C8—H8	119.1	C14—C19—H19	120.0
C7—C8—H8	119.1	C17—C20—H20A	109.5
C8—C9—C4	119.6 (3)	C17—C20—H20B	109.5
C8—C9—H9	120.2	H20A—C20—H20B	109.5
C4—C9—H9	120.2	C17—C20—H20C	109.5
C7—C10—H10A	109.5	H20A—C20—H20C	109.5
C7—C10—H10B	109.5	H20B—C20—H20C	109.5
			
C3—N1—N2—C4	−177.1 (3)	N2—C4—C9—C8	177.5 (4)
C13—N3—N4—C14	−176.5 (3)	N4—N3—C13—C12	178.9 (3)
N2—N1—C3—C2	−177.1 (3)	N4—N3—C13—Cl2	0.6 (5)
N2—N1—C3—Cl1	2.5 (5)	O2—C12—C13—N3	−179.3 (3)
O1—C2—C3—N1	175.7 (4)	C11—C12—C13—N3	0.7 (5)
C1—C2—C3—N1	−4.4 (5)	O2—C12—C13—Cl2	−0.9 (5)
O1—C2—C3—Cl1	−3.9 (5)	C11—C12—C13—Cl2	179.1 (3)
C1—C2—C3—Cl1	176.0 (3)	N3—N4—C14—C19	0.4 (5)
N1—N2—C4—C5	−172.5 (3)	N3—N4—C14—C15	179.8 (3)
N1—N2—C4—C9	8.7 (5)	C19—C14—C15—C16	1.9 (5)
C9—C4—C5—C6	1.3 (6)	N4—C14—C15—C16	−177.5 (3)
N2—C4—C5—C6	−177.6 (3)	C14—C15—C16—C17	−1.5 (6)
C4—C5—C6—C7	−0.4 (6)	C15—C16—C17—C18	0.0 (6)
C5—C6—C7—C8	−0.5 (6)	C15—C16—C17—C20	178.7 (4)
C5—C6—C7—C10	177.2 (4)	C16—C17—C18—C19	1.1 (5)
C6—C7—C8—C9	0.5 (6)	C20—C17—C18—C19	−177.6 (4)
C10—C7—C8—C9	−177.2 (4)	C17—C18—C19—C14	−0.7 (5)
C7—C8—C9—C4	0.5 (6)	C15—C14—C19—C18	−0.9 (5)
C5—C4—C9—C8	−1.3 (6)	N4—C14—C19—C18	178.5 (3)

### Hydrogen-bond geometry (Å, °)

**Table:**

*D*—H···*A*	*D*—H	H···*A*	*D*···*A*	*D*—H···*A*
N2—H2···O2	0.88 (5)	2.12 (5)	2.975 (4)	162 (4)
N4—H4···O1^i^	0.83 (5)	2.12 (5)	2.909 (4)	159 (4)

Symmetry codes: (i) *x*+1, *y*, *z*.
